# Three ways for art to stimulate the mind

**DOI:** 10.1080/17571472.2017.1333590

**Published:** 2017-06-05

**Authors:** Michael Drucquer

**Affiliations:** Study Day Organiser, Leicester, UK

**Keywords:** Medicine in arts

## Abstract

The relevance of the arts to medical practice is not always immediately apparent. Here, three examples are given from literature, visual arts and music which demonstrate links not normally found in medical texts.

**Why this matters to me – that is to you personally**

I enjoy discovering and hearing of surprising areas of overlap between the expressive arts and medicine and passing these on to others. Also, I find it fascinating and enlightening to observe how others experience and view the medical process, both in the past and present.

**Key message**

Expressive arts can articulate facets of medical practice which are otherwise hard to reach.

The last 20 years has seen a blossoming of interest in the medical humanities, with health professionals attracted to the study of patient experience and the medical world from perspectives other than the bio-medical. Sceptics may doubt the value of this, but I would maintain that the medical humanities, including the expressive arts as related to medicine, can contribute to reflective practice even when the areas studied might initially appear not to have a clear and overt medical content. Here are three examples where the subject matter’s linkage to medicine becomes progressively more tenuous, but does not disrupt the connection with the experiences of practitioners.

Take a novel like ‘Nemesis’ by Philip Roth [1]. Its overt medical content relates to a fictional polio epidemic in New Jersey in 1944 and its effects on individuals. Polio in the twentieth century is an exemplary case study of the interaction between public perception, panic responses, scientific blind alleys, scientific and epidemiological breakthroughs, competition amongst and between doctors and other health professionals and the interface between medicine, politics and mega-funding. Roth’s concise, rich, deceptively simple tour de force has as its main theme that of a male playground leader, Bucky, who cannot go to war because of short sight, who makes an error of judgement and cannot come to terms with it. Why not? Roth suggests that his background and context have made him over-conscientious, inflexible and prone to feelings of unworthiness, guilt and self-blame, even though he is honest, kind and personable. Sounds familiar? It will if you have read anything about precursors to burn-out or have experienced the dread feeling of recognising a mistake. The discussion our book group had about this novel demonstrated the ‘gateway’ aspect of art for medics, not only to the scientific and epidemiological context (for which ‘Paralysed with Fear, The Story of Polio’ by Gareth Williams [2] serves as an excellent companion read) but also as a stimulus to consider areas which we might otherwise find difficult to approach.

Moving on to the visual arts, take a look at Van Gogh’s ‘The Potato Eaters’ by entering this URL: https://www.vangoghmuseum.nl/en/collection/s0005V1962. It is considered to be Van Gogh’s first masterpiece and he took great time and effort over it. Three generations sit around a table sharing a dark coffee-like drink and a big platter of steaming potatoes. Grandad leans on his stick and proffers his cup for early seconds. The corner of the table leads the eye to the centrally placed back-facing daughter, suffused in a steamy glow. Check the composition and lighting; zoom in on the gnarled hands and the eyes of the mother. A drab picture at first sight, the people ugly, but something shines through. Where’s the medicine in this? It is revealed through this expression of the human condition, the companionship of family despite adversity, people grateful for small mercies. I have found that GPs and others who visit patients in their own homes appreciate such an artistically sympathetic representation of something they have often seen and are glad to be reminded not to lose sight of. If you are interested, you could also google Van Gogh’s letters/The potato eaters http://vangoghletters.org/vg/letters/let497/letter.html. It’s fascinating, but do you detect just a whiff of mania? Maybe it is a cliché to ask, but did that feed his genius?

Finally, the music of Elgar is often associated with Edwardian pomp and bombast, but more recently he has been re-evaluated. A self-taught Catholic ‘outsider’ of lowly origins he had to struggle for both recognition and income. Listeners now recognise the insecurities and uncertainties expressed in his music. As he said, his first symphony ‘was written out of a full life experience and is meant to include the innumerable phases of joy and sorrow, struggle and conquest, and especially between the ideal and the actual life.’ I’m no classical music buff, but I have been educated in Elgar by one of our ‘arts for medicine’ delegates, for which thanks are due. Try Elgar’s Symphony No 1 in A flat major (2nd movement if short of time) – you will pick up on the light and shade contrasts, the calm alternating with agitation, and you will be reminded of parallels with both illness and the joys and tribulations of medical practice. You can listen on Youtube at https://www.youtube.com/watch?v=sCuSuwDXxUA.

None of the above examples are, on the face of it, about doctors, patients or specific illness experience. However, I would maintain that each example can provide insights which will resonate with us even though the pertinence may initially seem obscure. When viewed as a linkage to the topics of clinician error, the unique value of home visits or the emotional highs and lows of an ‘outsider’, we can see that they articulate at a nuanced, imaginative and non-descriptive level, our common experience of the practice of medicine.

## Disclosure statement

No potential conflict of interest was reported by the author.
